# Doctors

**Published:** 1876-04

**Authors:** 


### DOCTORS.

DR. NED.

The first account we have of doctors is found in the book of Genesis, where it is stated that when Jacob died, "Joseph commanded his servants, the physicians, to embalm him." This occurred 1700 years prior to the birth of Christ. The first physician of whom we have any information, was Doct. Moses, who was found by Miss Pharaoh, and educated after the manner of the Egyptian priesthood, who had up to this time done all the professional business of the country, and under their laws, the profession had been greatly checked in its progress, for they had a statute which provided that if any doctor departed from the regular practice laid down by their school, and failed to save the life of his patient, he was punished by death; and under such circumstances, of course, the profession could not make much headway, for no one would dare institute an experiment unless he had first caused all the stones in the country to be picked up and removed, for he was certain, in those days, if he failed, to be investigated, and he could expect nothing better than a shower of rocks going for him at the rate of a thousand miles a minute, just as soon as he could get outside of the city limits, where they were allowed to hurl their deadly missiles. It is wonderful how those old people could throw stones; the inhabitants of the Emerald Isle could not compare with them.—I am glad this destructive system of warfare has given place to those less dangerous agents, powder and balls. Those who are very fond of medical ethics, and willing to submit without a murmer to the arbitrary rules of medical societies, which insist upon the right of thinking for all their subjects, will be glad to know that these organizations are of ancient origin, and that the Fathers "peddled pills" among the Egyptian scholars.

Doct. Moses is certainly entitled to great credit and veneration, from the fact that he was the first to pay any attention to the very important matter of dietetics, and fixed out a "bill of fare," which, with but a few exceptions, is still in vogue. He prohibited eating rabbits and swine, which are now indulged in by the masses in defiance of the instructions of the Doctor. As far as the former is concerned, I am inclined to think the Doctor was incorrect, but on the hog question there is no doubt he was sound, for I contend that there is no such thing as a healthy "porker." You may keep one as clean as possible, and feed him on the very best provisions, wash him every day, comb his hair, wipe his face, brush his whiskers and wax his moustache, let him out of a parlor and he will jump in the first mud hole he can find, and has the itch so bad that he will rub the hind wheel off from a lumber wagon in twenty minutes, by a stem winder watch; and within six years, the brute will die of phthisis pulmonalis. I tell you the nail was hit on the head for once, and with Doct. Moses I am ready to exclaim, "not any hog for me." Some of our ablest authors and writers adhere to the old idea, and to this day ignore the rabbit, as taught by the venerable Doct. M., and agree with our eminent philosopher, Mr. Billings, who says, "the rabbit iz a kind ov long-eared and short-taled rat with hind legs twice az long an twice az fast az his 4 ones," and that they seem to be "bilt for running up a hill and backing down it," and he also remarks that they "can hatch out and spred faster than the meazles," and respecting their eating qualities, he says that "they bile easy, eat soft, and are sed tew be better vittles than the kat." It may not be generally known, but it is nevertheless true, that Doct. Moses, by his minute description of the leprosy, placed himself in the front ranks among medical diagnosticians. He gives us all the symptoms of this disease and its treatment, and does not, like many of our modern physicians, waste any time speculating upon the causes which produced it.

Of late much has been said and done about the matter of destroying the contagion of certain diseases which clings to inanimate objects. We talk of fumigating houses, &c., just as though it was a new thing, when in fact, the profession is under obligations to Doct. M. for the discovery, for in his day, if any infectious disease occurred in a house, he ordered it plastered anew and perfectly cleansed, and if that did not answer the purpose, he would not waste any more time upon it, or endanger any more people, but he at once ordered that shanty pulled down and carried out of town. We hear much said of one Hannemann, who less than a century since claimed to have discovered the principle of "similia similibus curanter," as well as the secret of curing diseases with doses of medicine so small that they could neither be seen or weighed. If there is any truth in this law of cure, or effectiveness in such quantities, the credit of the discovery belongs to Doct. Moses, who directed all that were bitten by the fiery serpents, just to take a look at the one he had made of brass and hoisted upon a pole; and it is said that he cured every case. Doct. Moses at the conclusion of his professional labors, promulgated to his people the Decalogue, and from that time for a considerable period, Doct. Solomon occupied the front ranks among men of science and learning. John Thompson and his followers, who profess to treat all diseases with roots and herbs, arrogate to themselves great merit in having discovered the healing properties of vegetable products, and in this way exhibit a selfishness that seems to be peculiar to all schools of medicine. The truth of the matter is, that Doct. Solomon, who spoke five hundred proverbs and wrote three thousand songs, was the first botanic physician of whom any record is made; he spoke of "plants from the cedar of Lebanon, even unto the hyssop that springeth out of the wall." And Josephus tells us that this Doctor has a "perfect knowledge of the properties of all the productions of nature," and that some of his remedies "had even the virtue to cast out devils." It is a great pity that the Doctor did not write and transmit to posterity a materia medica. It would have saved an immense amount of labor and still more guessing, and besides all that, no one can tell what the demand at this day would have been for the "devilish" compound of which we know nothing, and we are therefore obliged to place this valuable remedy among the lost arts and leave it to be speculated upon by modern Phillips.

Leaving Doctors Moses and Solomon, as well as many others who flourished in about that time, we come to the Oriental Indians who had a system of medicine peculiar to themselves. They thought the human body was made up of one hundred thousand parts, of which seventeen thousand were vessels, composed each of seven tubes, giving passage to ten species of gasses, engendering in this way a crowd of diseases that no man could number or understand. They consulted the sun, moon and stars; they claimed that the pulse originated from a great reservoir located in the region of the bowels, and that the gift of healing was of a divine origin and came direct from *above*. They minutely examined everything but the patient, and looked wise and mysterious about everything that could not have the least bearing upon the case, and prescribed accordingly. And, what is wonderful, they were driven with business all the while; the people in that early day had a taste for humbuggery, which has evidently been handed down to this generation. The proof can be found in almost any of our towns or cities where you will see the signs of "Indian Doctors," "Chinese Physician," "Clairvoyant Treatment," etc., etc., and to those impostors and ignoramuses thousands of our people flock, to be cured of all the "ills flesh is heir to." Such men have neither education, experience or brains: they have as a rule neither responsibility, character or caution, and yet they are trusted with human life. The less they know the better it would seem, and I have often thought, and *now* think, that if Baalam's mule could turn up and hang out "a shingle" that the brute would do more business in ten years than any honorable cultivated physician in the country. This humbuggery should be cultivated, for it shows beyond dispute that the intellectual condition of society is on the advance. And besides, it is an extra inducement to our young men to study year after year, that they may be fitted to grapple with disease, and then have a first-class chance to starve, while some "big Indian," or "Great English Physician," with a gilded lackey at his heels, to add dignity and importance to his distinguished presence, or John Chinaman, with a pig tail nine feet long, get public patronage, and place hosts of curable cases beyond the reach of human skill. They diagnose disease by examining a lock of the hair, or an eye tooth, or a dirty stocking. They expel tape worms "all cut to pieces," with heads as large as a family bible and tails longer than a modern telegraph pole; they coax toads, snakes, alligators, fish, rats, mice, kittens and small dogs, to come out of human stomachs, for a meal of bologna sausages or limberger cheese; they cure cancer with a plaster; rheumatism, gout, gravel, headache and corns they charm away, by looking directly east and making nine passes on the right side and eleven on the left, at two dollars each, and pay in advance, and in this way people are duped, and talent, energy and enterprise encouraged in our country. This surely is an age of progress.

